# Depth-Enhanced Holographic Super Multi-View Maxwellian Display Based on Variable Filter Aperture

**DOI:** 10.3390/mi14061167

**Published:** 2023-05-31

**Authors:** Kefeng Tu, Qiyang Chen, Zi Wang, Guoqiang Lv, Qibin Feng

**Affiliations:** 1 School of Instrument Science and Opto-Electronics Engineering, Hefei University of Technology, Hefei 230009, China; kefengtu@mail.hfut.edu.cn (K.T.); tseeyung@mail.hfut.edu.cn (Q.C.); fengqibin@hfut.edu.cn (Q.F.); 2 National Engineering Laboratory of Special Display Technology, National Key Laboratory of Advanced Display Technology, Academy of Photoelectric Technology, Hefei University of Technology, Hefei 230009, China; guoqianglv@hfut.edu.cn

**Keywords:** near-eye display, three-dimensional display, holographic display, super multiview, depth of field

## Abstract

The super multi-view (SMV) near-eye display (NED) effectively provides depth cues for three-dimensional (3D) displays by projecting multiple viewpoint images or parallax images onto the retina simultaneously. Previous SMV NED suffers from a limited depth of field (DOF) due to the fixed image plane. Aperture filtering is widely used to enhance the DOF; however, an invariably sized aperture may have opposite effects on objects with different reconstruction depths. In this paper, a holographic SMV display based on the variable filter aperture is proposed to enhance the DOF. In parallax image acquisition, multiple groups of parallax images, each group recording a part of the 3D scene on a fixed depth range, are captured first. In the hologram calculation, each group of wavefronts at the image recording plane (IRP) is calculated by multiplying the parallax images with the corresponding spherical wave phase. Then, they are propagated to the pupil plane and multiplied by the corresponding aperture filter function. The size of the filter aperture is variable which is determined by the depth of the object. Finally, the complex amplitudes at the pupil plane are back-propagated to the holographic plane and added together to form the DOF-enhanced hologram. Simulation and experimental results verify the proposed method could improve the DOF of holographic SMV display, which will contribute to the application of 3D NED.

## 1. Introduction

The near-eye display is a promising technology which enables virtual reality (VR) and augmented reality (AR) [1,2,3]. Binocular display technology is widely used in three-dimensional (3D) NEDs. However, this type of display suffers from the vergence–accommodation conflict (VAC), leading to visual fatigue and discomfort [4]. Various kinds of methods have been proposed to relieve the VAC problem, such as light field display [5,6,7,8,9], multi/varifocal displays [10,11,12], and holographic displays [13,14,15,16,17,18,19,20,21,22,23]. These techniques reconstruct the optical field to provide accurate depth cues and have demonstrated impressive results. However, the matched video sources are still hard to access or generate for commercial use due to the large amount of 3D data. Apart from these, the Maxwellian NED is less computationally expensive and relieves the VAC problem by providing an “always in-focus” image, but the monocular depth cues are not recovered [24,25,26,27,28,29,30,31,32].

SMV NEDs provide depth cues to the viewer by projecting multiple viewpoint images or parallax images into the pupil simultaneously [33,34,35,36,37,38,39]. Since two or more viewpoints exist in the pupil, two or more rays passing through one point of a 3D image enter the pupil simultaneously through the viewpoints, which induces eye lens focus on that point and provides a correct accommodation depth cue. SMV NED is less computationally demanding because it only needs to calculate the parallax image information. The parallax image generation technology is mature now and the existing parallax image resource is abundant which makes it easy to achieve commercial applications.

Depth of field (DOF) is an important parameter in SMV displays which determines the extent to which 3D images can be rendered clearly. The width of the light ray produced by a single-object pixel determines the DOF. The narrower the light ray is, the larger the DOF will be. To increase the DOF, an effective method is to limit the width of the light beam entering the pupil. The light-emitting diode (LED) light sources were usually used and collimated to illuminate the spatial light modulator (SLM) [33,34,35]. The finite size of the LED source influenced the DOF of the system. In Ref. [37], an SMV Maxwellian display based on a collimated laser source is proposed where the limited light ray enhances the DOF. The SMV Maxwellian display is a mixture of the SMV display and the Maxwellian display. The Maxwellian display converges the light ray into the pupil which enlarges the DOF of the SMV display due to its small exit pupil. In Ref. [38], a holographic SMV Maxwellian display was used to improve the DOF of the SMV display. This method has simple eyebox expansion and little lens aberration by wavefront modulation. The Maxwellian display does not mean that it has an infinite DOF. It also has one fixed virtual image plane, and its DOF range exists around this image plane. When the 3D image gets farther away from the image plane, its image quality still gets worse. Although numerical aperture filtering could be used to limit the light ray width to enhance the DOF, the image quality degrades in turn due to the loss of high-frequency components [40]. That is, there is a trade-off between the image quality and DOF.

In this paper, a holographic SMV Maxwellian display based on variable filter aperture is proposed to enhance the DOF. Objects at different depths are captured as different groups of parallax images first. In the hologram calculation, each group of wavefronts at IRP is calculated by multiplying the parallax image with the corresponding spherical wave phase. Then, they are propagated to the pupil plane and multiplied by the corresponding aperture filter. In our method, based on the analysis of filter aperture size and DOF, filter aperture size was variable according to the depth of the reconstructed object. Finally, the complex amplitudes at the pupil plane are back-propagated to the holographic plane and added together to form the DOF-enhanced hologram. Simulation and experimental results verify that the proposed method could improve the reconstruction quality of objects far from the IRP plane and maintain the reconstruction quality of image around the IRP plane which enhance the DOF of holographic SMV display.

## 2. The Limited DOF for Conventional SMV Maxwellian Display

**Figure 1:**
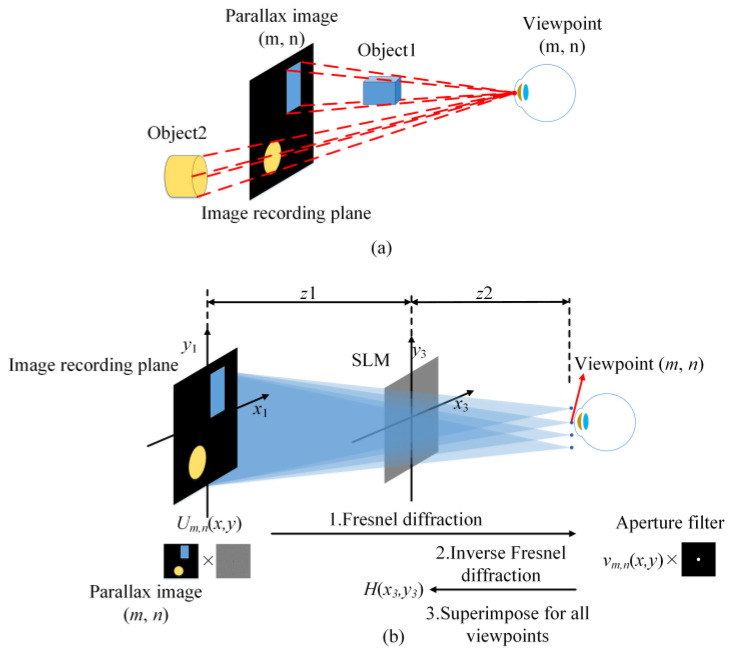
(**a**) Acquire the parallax image of viewpoint (*m*, *n*); (**b**) Conventional Fresnel diffraction calculation of holographic RPD.

Figure 1 shows the principle of the typical holographic SMV Maxwellian display. Two or more parallax images are simultaneously converged to the pupil. We assume that a total of *M* × *N* viewpoints are set, and the corresponding parallax images of 3D objects are prepared in advance as shown in Figure 1a. The SMV Maxwellian hologram can be obtained by four steps as shown in Figure 1b. Firstly, the complex amplitude distribution of the corresponding parallax image (*m*, *n*) on the image plane is obtained by multiplying the parallax image amplitude with a spherical wave converging to the viewpoint (*m*, *n*).

(1) $$U_{m,n}(x_{1},y_{1})=A_{m,n}(x_{1},y_{1})\cdot \exp\left\{\frac{-jk\left[{\left(x-x_{m}\right)}^{2}+{\left(y-y_{n}\right)}^{2}\right]}{z_{1}+z_{2}}\right\},$$

where *k* = 2π/λ is the wavenumber, *z*_1_ is the distance from the target image to the SLM, and *z*_2_ is the distance from the SLM to the pupil plane. Secondly, the complex amplitude distribution *v*_m,n_(*x*_2_,*y*_2_) on the pupil plane is calculated through a Fresnel diffraction as shown in Equation (2). To avoid the possible crosstalk between adjacent viewpoints, the complex amplitude distribution *v_m_*_,*n*_(*x*_2_,*y*_2_) on the pupil plane is multiplied by the aperture filter function corresponding to the viewpoint as shown in Equation (3).

(2) $$v_{m,n}(x_{2},y_{2})=\iint U_{m,n}(x_{1},y_{1})\cdot \exp\left\{\frac{jk\left[{\left(x-x_{2}\right)}^{2}+{\left(y-y_{2}\right)}^{2}\right]}{2(z_{1}+z_{2})}\right\}dxdy,$$

(3) $$V_{m,n}(x_{2},y_{2})=v_{m,n}(x_{2},y_{2})\cdot circ(\frac{\sqrt{{\left(x_{m}-p/2\right)}^{2}+{\left(y_{n}-p/2\right)}^{2}}}{p/2}),$$

where circ() represents a circular aperture with (*x*_m_, *y*_n_) as its center and *p*/2 is the radius of the circle. Thirdly, the complex amplitude *V_m_*_,*n*_ in the pupil plane is propagated backwards to the holographic plane to obtain the hologram of the viewpoint (*m*, *n*). The final SMV Maxwellian hologram is obtained by superimposing the holograms of each viewpoint as shown in Equation (4). The reconstructed image can be observed when the encoded hologram is loaded in the spatial light modulator.

(4) $$H(x_{3},y_{3})=\sum_{m=1}^{M}\sum_{n=1}^{N}\iint V_{m,n}(x,y)\cdot \exp\left\{\frac{jk\left[{\left(x-x_{3}\right)}^{2}+{\left(y-y_{3}\right)}^{2}\right]}{-2z_{2}}\right\}dxdy.$$

**Figure 2:**
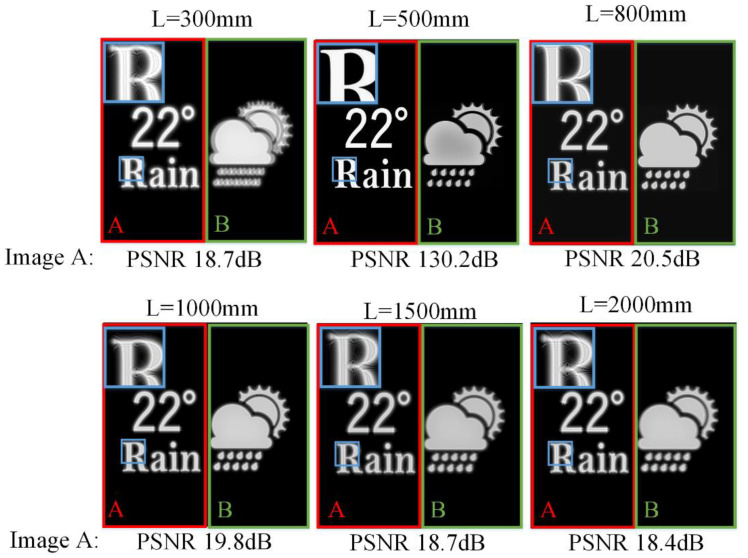
The reconstructed images of conventional holographic SMV Maxwellian display at different depths.

Figure 2 shows the simulated reconstruction results at different reconstruction depths *L* of holographic SMV Maxwellian display. Two viewpoints are set at the pupil plane. Images A and B are located at different depths. Image A is at the reconstruction depth *L*. Image B is always at 0.8 m from the human eye. The parameters are set as: Δ*x* = 12 µm, *N* = 4096, *z*_1_ = 360 mm, *z*_2_ = 140 mm, *λ* = 532 nm, where *N* is the resolution of the target image. The image-recording plane (IRP) is 0.5 m away from the human eye.

Image B becomes ghosted and blurred when the depth of the reconstructed image is far from the depth of image B. The out-of-focus phenomenon provides depth clues for monocular observation. Image A is always in the reconstruction plane, and thus it should be in focus. However, image A is blurred when the reconstructed depth is far from the IRP. The limited DOF of the IRP affects the quality of the reconstructed image and provides incorrect depth clues for monocular observation.

**Figure 3:**
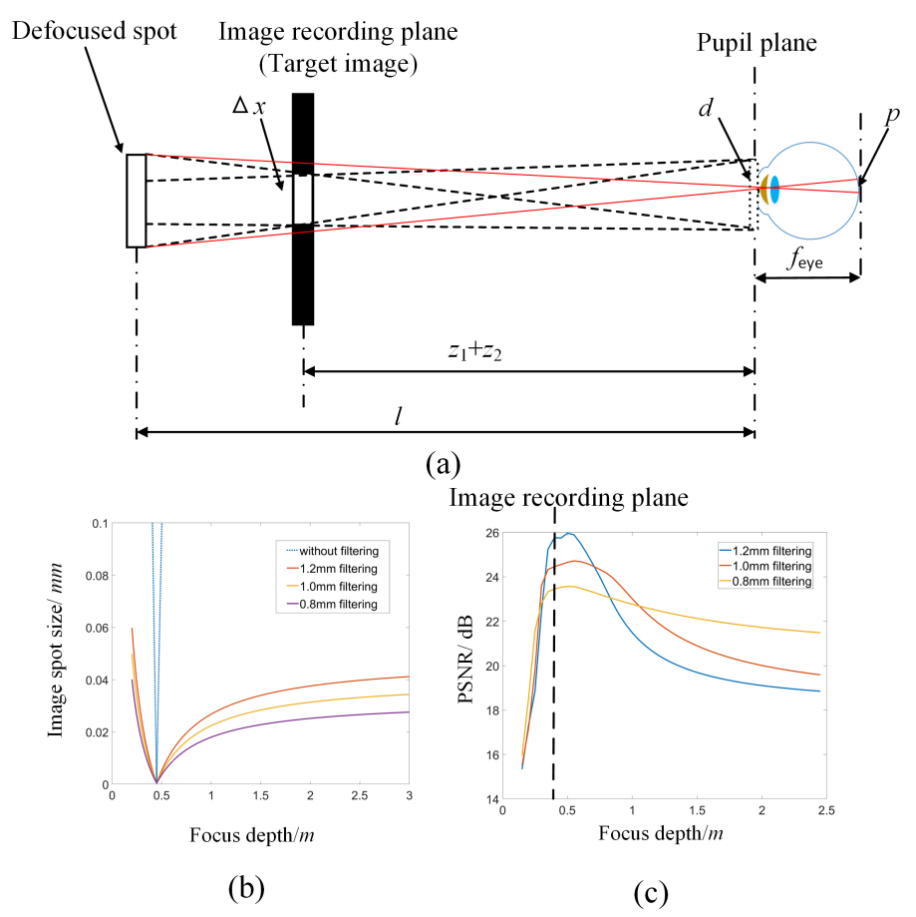
(**a**) The process of imaging a pixel on the IRP to the retina. (**b**) The size of a pixel on the retina at different reconstruction depths. (**c**) The PSNR of reconstructed images at different depths.

The maximum acceptable spot width *δ* on the retina is used to calculate the DOF of the SMV Maxwellian display. If the image pixel size *p* on the retina is larger than *δ*, the image will no longer be clear. The DOF of the SMV Maxwellian display depends on the DOF of each viewpoint. Thus, we use a single-viewpoint Maxwellian display model to calculate the DOF. The spot width of a target image pixel in the pupil plane *d* is given by Equation (5).

(5) $$d=\frac{\lambda (z_{1}+z_{2})}{\Delta x},$$

where Δ*x* is the pixel size of the target image. According to Figure 3a and Equation (5), the maximum image spot size *p* of one pixel of the target image on the retina is given as:

(6) $$p=\left(\Delta x\frac{l}{z_{1}+z_{2}}+\frac{\left|z_{1}+z_{2}-l\right|}{z_{1}+z_{2}}d\right)\frac{f_{eye}}{l}\approx \frac{\left|z_{1}+z_{2}-l\right|\cdot d\cdot f_{eye}}{\left(z_{1}+z_{2}\right)l},$$

Due to Δ*x* as a minimal value, the relevant term is ignored.

To better show the DOF of the target image, numerical simulation for the change of *p* at different focus depths is performed. The parameters are set as: Δ*x* = 0.1 mm, *z*_1_ = 300 mm, *z*_2_ = 150 mm, *λ* = 532 nm, *f*_eye_ = 18 mm. The DOF of the image can be obtained according to the maximum acceptable spot width δ and curve of *p.* As shown in Figure 3b, the spot size of the reconstructed image on the retina increases sharply when the depth of the reconstructed image is far from the image recording plane. Aperture filtering on the pupil plane can be applied to reduce the spot width *d* for DOF enhancement. However, aperture filtering loses part of the high-frequency information, which will cause image degradation. Figure 3c shows the PSNR of the image at different reconstruction depths under different aperture filtering. It is found that for the reconstruction near the IRP, a small aperture filter size will degrade the image quality. However, for the reconstruction far away from the IRP, a small aperture size will improve the image quality. Thus, the invariable aperture may have opposing effects on the reconstruction at different depths.

## 3. Variable Filter Aperture for SMV Maxwellian Display

**Figure 4:**
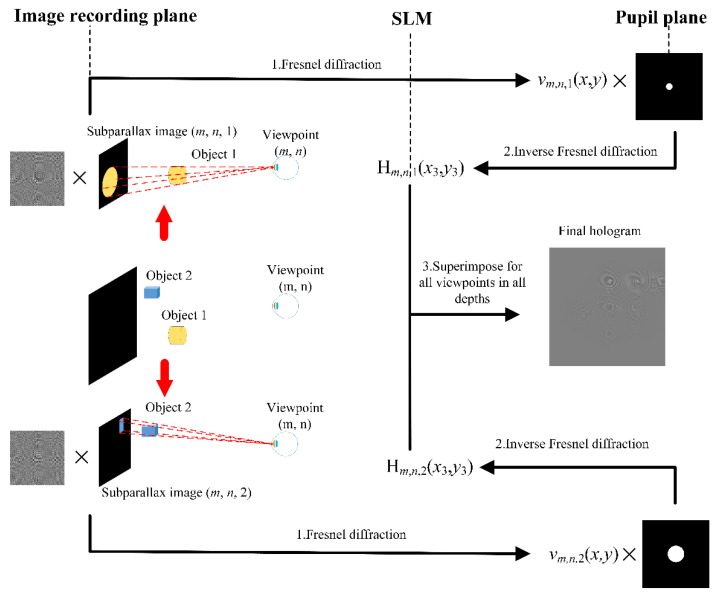
The principle of variable filter aperture-based SMV Maxwellian display.

Based on the analysis of DOF, to improve the quality of the image at each reconstruction depth, a variable filter aperture method is proposed. Figure 4 shows the principle of variable filter aperture for SMV holographic Maxwellian display. In parallax image capture, as shown in Figure 4, parallax images of objects at different depths are acquired independently. The process of acquiring the hologram can be divided into four steps which are the same as the conventional holographic SMV Maxwellian display. However, there is a difference in the filter aperture at the pupil plane between these two methods. In the conventional holographic SMV Maxwellian display, the filter aperture size is fixed for objects at different reconstruction depths. In our proposed method, parallax images of objects at different depths are multiplied by the corresponding aperture filter functions in generating holograms. The image pixel size *p* on the retina in Eq. 6 determines the size of the filter aperture. When the object is farther away from the IRP, the filter aperture needs to be smaller to limit the size of the image pixel *p* on the retina. Thus, the filter aperture *d_p_* is given by:

(7) $$d_{p}=p\frac{(z_{1}+z_{2})\cdot l}{\left|z_{1}+z_{2}-l\right|\cdot f_{eye}},$$

**Figure 5:**
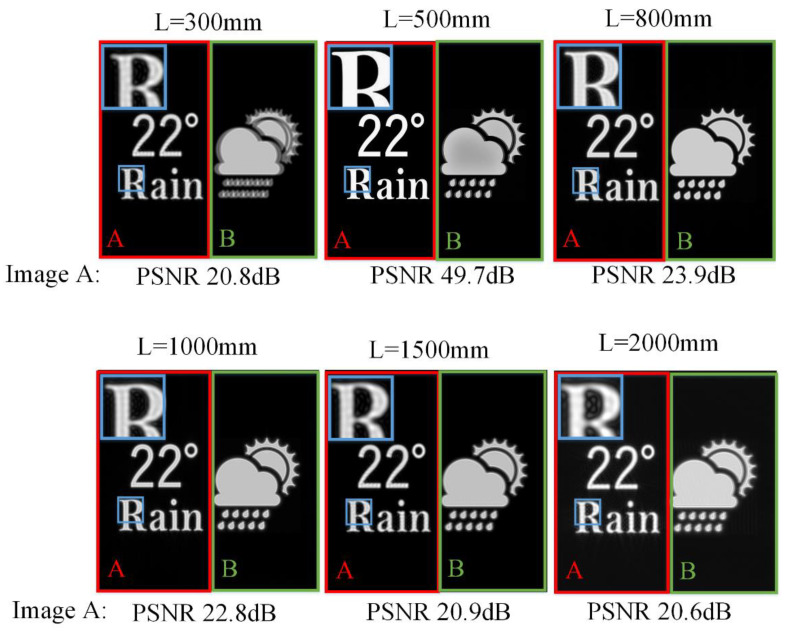
The reconstructed images of variable filter aperture-based SMV Maxwellian display at different depths.

Figure 5 shows the simulation results using the proposed method. The parameter settings are the same as the simulation of the conventional SMV Maxwellian display. When image A is 300, 800, 1000, 1500, 2000 mm away from the pupil plane, the filter aperture is 0.625, 1.111, 0.833, 0.625, 0.556 mm, respectively. Filtering is not performed when image A is in the parallax image recording plane. Image B is always at 800 mm from the pupil plane, thus the filter aperture is kept constant at 1.111 mm. Compared to the simulation results of conventional SMV Maxwellian display, the PSNR of reconstructed images at 5 reconstruction depths is improved by 2.58 dB on average which demonstrated the variable aperture-filtered SMV has improved DOF. Although the reconstructed image at IRP is affected by other depth filtering, the PSNR is kept above 40 dB which is difficult for the human eye to observe the difference.

**Figure 6:**
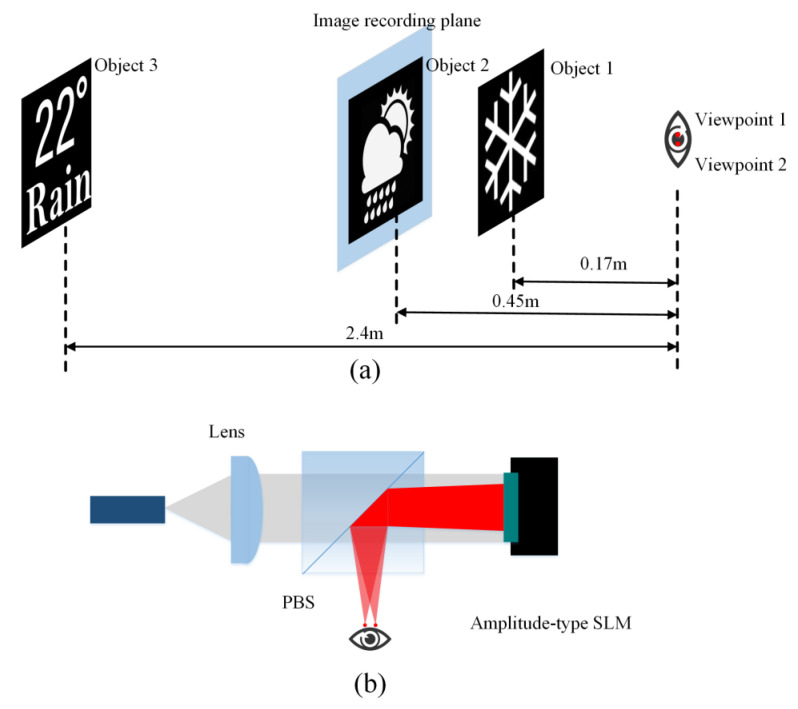
(**a**) The location of the object and the IRP; (**b**) The experiment setup.

**Figure 7:**
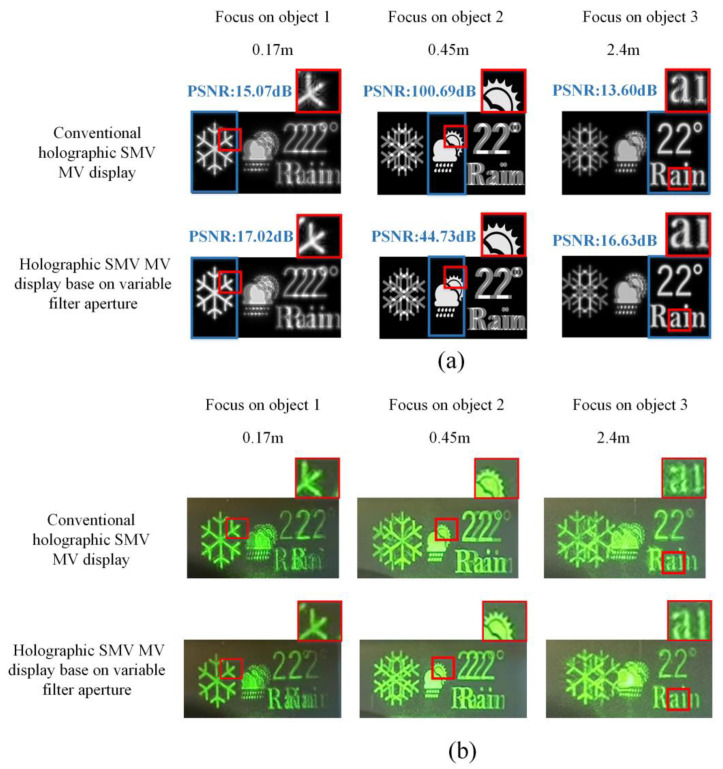
The comparison of the (**a**) simulation results (**b**) experimental results between the conventional SMV Maxwellian display and the proposed method at different reconstruction depths.

The simulation and experiment are presented to prove the proposed method. As shown in Figure 6a, three objects were located 0.17 m, 0.45 m, and 2.4 m from the viewpoint, respectively. According to Equation (7), the filter aperture is 0.23 mm for object 1 and 0.46 mm for object 3. The IRP is at the same position as object 2. Thus, object 2 is not filtered. For simplicity, two viewpoints are set at the pupil plane. The distance between the two viewpoints is 1.15 mm. Figure 6b shows the experimental setup. A laser beam with 532 nm wavelength was collimated by the lens and illuminated the SLM after passing the polarization beam splitter (PBS). An amplitude-type spatial light modulator (SLM) (3.6 µm pixel pitch, 4096 × 2160 resolution) was used to load the hologram. The modulated s-polarized light was reflected by the PBS and entered the pupil. Simulation and experimental results were demonstrated in Figure 7. The variable filter aperture method enhances the image quality at the reconstruction depth of object 1 and object 3. The reconstructed image of object 2 maintains high quality. In addition, the eye box of the RPD could be expanded by duplicating viewpoints at the pupil plane [39].

## 4. Conclusions

To enhance holographic SMV displays’ limited DOF, a holographic SMV display based on variable filter aperture is proposed. Different-sized filter apertures are applied for objects in different depths to improve the reconstruction quality at each depth. Firstly, multiple groups of parallax images are captured. Each group recorded a part of the 3D scene on a fixed depth range. Each group of wavefronts at IRP is calculated by multiplying the parallax images with the corresponding spherical wave phase. Then, they are propagated to the pupil plane and filtered with a different aperture. The size of the aperture is variable which is determined by the depth of the object. The final DOF-enhanced hologram is obtained by back-propagating the complex amplitude in the pupil plane to the holographic plane and adding together. Simulation and experimental results verify the proposed method could improve the DOF of holographic SMV display. The method is computationally simple and easy to access which is promising for realizing VAC-free 3D NED with large DOF.

## Data Availability

Data are contained within the article.
